# Hip Position Acutely Affects Oxygenation and Perfusion of Kidney Grafts as Measured by Functional Magnetic Resonance Imaging Methods—The Bent Knee Study

**DOI:** 10.3389/fmed.2021.697055

**Published:** 2021-08-10

**Authors:** Laila-Yasmin Mani, Maryam Seif, Florence Nikles, Dechen W. Tshering Vogel, Gaëlle Diserens, Petros Martirosian, Michel Burnier, Bruno Vogt, Peter Vermathen

**Affiliations:** ^1^Department of Nephrology and Hypertension, Inselspital, Bern University Hospital, University of Bern, Bern, Switzerland; ^2^Departments of Biomedical Research and Radiology, University of Bern, Bern, Switzerland; ^3^Spinal Cord Injury Center, Balgrist University Hospital, University of Zurich, Zurich, Switzerland; ^4^Department of Diagnostic, Interventional and Pediatric Radiology, Inselspital, Bern University Hospital, University of Bern, Bern, Switzerland; ^5^Section on Experimental Radiology, University of Tübingen, Tübingen, Germany; ^6^Service of Nephrology and Hypertension, Department of Medicine, Lausanne University Hospital, Lausanne, Switzerland

**Keywords:** hip flexion, kidney transplantation, perfusion, oxygenation, functional MRI, BOLD, arterial spin labeling, multiparametric magnetic resonance imaging

## Abstract

**Background:** Kidney perfusion and oxygenation are two important determinants of kidney graft function. In kidney transplantation, repeated graft hypoperfusion may occur during hip flexion, for example in the sitting position, due to the progressive development of fibrotic tissue around iliac arteries. The aim of this study was to assess the changes in oxygenation and perfusion of kidney grafts during hip flexion and extension using a new functional magnetic resonance imaging (fMRI) protocol.

**Methods:** Nineteen kidney graft recipients prospectively underwent MRI on a 3T scanner including diffusion-weighted, blood oxygenation level dependent (BOLD), and arterial spin labeling sequences in hip positions 0° and >90° before and after intravenous administration of 20 mg furosemide.

**Results:** Unexpectedly, graft perfusion values were significantly higher in flexed compared to neutral hip position. Main diffusion-derived parameters were not affected by hip position. BOLD-derived cortico-medullary R2^*^ ratio was significantly modified during hip flexion suggesting an intrarenal redistribution of the oxygenation in favor of the medulla and to the detriment of the cortex. Furthermore, the increase in medullary oxygenation induced by furosemide was significantly blunted during hip flexion (*p* < 0.001).

**Conclusion:** Hip flexion has an acute impact on perfusion and tissue oxygenation in kidney grafts. Whether these position-dependent changes affect the long-term function and outcome of kidney transplants needs further investigation.

## Introduction

Kidney transplantation is the therapy of choice for patients with end-stage kidney disease conferring survival benefit regardless of graft source (1). However, long-term graft survival has failed to improve in recent decades despite the great reduction in acute rejection episodes achieved by current immunosuppressive regimens (2). A major cause is the progressive interstitial fibrosis and tubular atrophy (IFTA) of the organ considered multifactorial in origin (3, 4).

In spite of advanced operative techniques, vascular complications after kidney transplantation remain of concern (5, 6). Surgical re-interventions are generally complicated by the marked fibrotic peri-graft reaction developing in the early post-transplant period (7).

Intriguingly, lower extremity claudication has been reported in professional cyclists due to kinking of iliac arteries during hip flexion visualized by magnetic resonance (MR) angiography (8, 9). Kinking was caused by tethering of iliac arteries by psoas or other arterial side branches, by fibrous fixation of the iliac bifurcation, or by chronic arterial stretching during repeated hip hyperflexion (10, 11). The claudication responded well to surgical release of the artery (9). In healthy subjects, maximal hip flexion has been shown to induce shortening, bending, and twisting of iliac arteries (12).

Little is known about dynamic perfusion changes in kidney grafts depending on body posture. Kidney grafts typically placed into iliac fossa might be exposed to similar position-dependent perfusion problems. Specifically, kinking or narrowing of the transplant renal or iliac artery may occur during hip flexion due to tethering by adjacent fibrotic tissue leading to iterative hypoperfusion episodes as well as chronic ischemic graft damage and IFTA in the long-term. Considering common sedentary lifestyle majorly in the sitting position, this influence could be significant. Furthermore, pre-existing endovascular lesions in this high-cardiovascular-risk population or subclinical vascular anastomotic problems may have an additional effect. To the best of our knowledge, this question has not been addressed so far.

Today, novel functional MR imaging (fMRI) techniques allow for the non-invasive investigation of renal tissue oxygenation, perfusion, and diffusion with the use of blood oxygenation level dependent (BOLD)-MRI, arterial spin labeling (ASL)-MRI, and diffusion-weighted imaging (DWI) (13).

The aim of this prospective interventional study was therefore to assess the influence of hip flexion on kidney graft oxygenation and perfusion using fMRI techniques in kidney transplant recipients. Our hypothesis was that hip flexion >90° (as achieved in the usual static sitting position) would lead to an instant and temporary reduction of renal tissue oxygenation and perfusion as measured by BOLD-MRI, ASL-MRI, and DWI compared to neutral hip position. To correlate results with vascular anatomy (presence of functional kinking and/or pre-existing endovascular lesions), non-contrast-enhanced time-of-flight (TOF) angiography during hip flexion and duplex-ultrasound scans (DUS) were performed. Additionally, we analyzed the correlation of oxygenation and perfusion changes with clinical parameters.

## Materials and Methods

The protocol of this prospective single-center interventional study was approved by the local ethics committee (Canton of Bern, Switzerland, protocol number 2181, approval number 042/12) and conducted in accordance with the Declarations of Helsinki and Istanbul (14, 15).

### Study Population

Patients ≥18 years having received a kidney graft ≥6 months ago into the iliac fossa with a stable graft function (≤ 30% deviation of last three serum creatinine values) and an estimated glomerular filtration rate (eGFR) ≥30 ml/min/1.73 m^2^ according to Chronic Kidney Disease Epidemiology Collaboration (CKD-EPI)-equation and preserved faculties of judgment were eligible.

Exclusion criteria were pregnancy; New York Heart Association stage IV dyspnea or orthopnea; acute infection; active neoplasia; surgical intervention, severe trauma, or acute ischemic or thromboembolic event within the preceding 2 months; inability of ipsilateral hip flexion; classical contraindications to MRI; body weight >200 kg; as well as any implanted metallic material without prior 3T-MRI after implantation.

### Study Design

From June 2015 through January 2016, 97 consecutive patients were screened at the outpatient University Clinic for Nephrology and Hypertension in Bern, 19 of whom fulfilled eligibility criteria and accepted to participate. Written informed consent was obtained from each participant prior to inclusion. A standardized hydration protocol was followed (2 L water intake over the preceding day; on the study day, 5 ml/kg upon awakening, followed by 3 ml/kg/h) and diuretics were held on the study day and the preceding day if considered safe by the treating nephrologist (16, 17). A light meal was allowed on the study day. Baseline clinical characteristics were obtained based on medical record review and blood was drawn for serum creatinine analysis.

**Figure 1:**
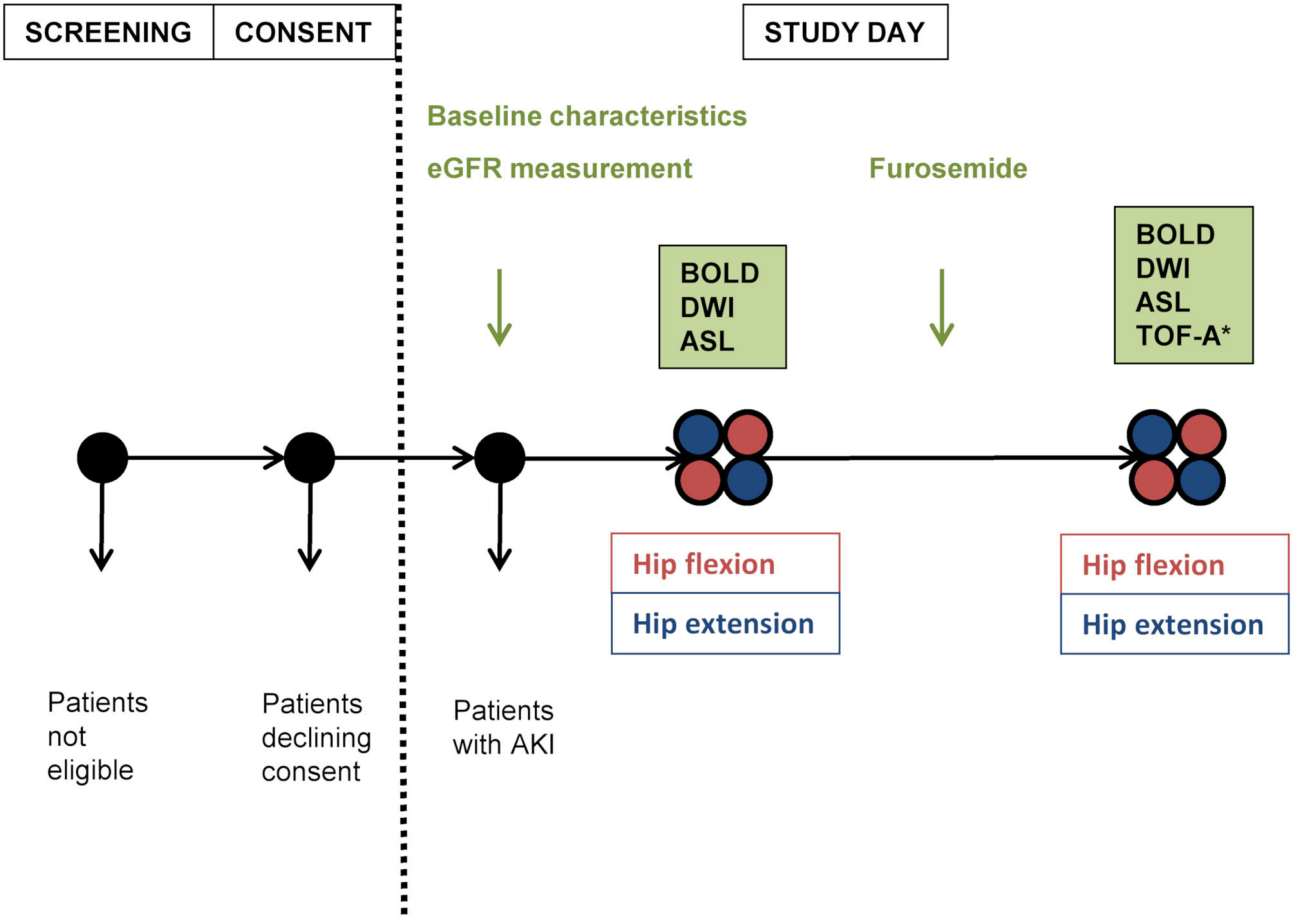
Study plan. Positioning order was alternated in each following subject, then maintained for the second part of measurements. eGFR, estimated glomerular filtration rate estimated according to chronic kidney disease epidemiology formula; AKI, acute kidney injury; BOLD, blood oxygen level-dependent magnetic resonance imaging; DWI, diffusion-weighted imaging; ASL, arterial spin labeling-magnetic resonance imaging; TOF-A, time of flight-angiography; ^*^performed during hip flexion.

There were four study phases during one patient's session: anatomical MRI, DWI, BOLD-MRI, and ASL-MRI were first performed during neutral hip position and then repeated during maximally achievable hip flexion (≥90° from bed level); to account for position-induced confounding effects on the BOLD signal as well as to include a functional test, the same measurements were repeated 10 min after the intravenous administration of 20 mg furosemide (LASIX®; Sanofi-Aventis, Vernier, Switzerland) (17–21). At the end of the last study phase, TOF angiography was performed during hip flexion. In order to minimize bias, the sequence of positions was alternated in each consecutive subject but maintained unchanged after furosemide administration in each subject (Figure 1).

### MRI Protocol

MRI data were acquired on a 3.0 T whole body MR Scanner (Magnetom Verio®; Siemens Healthcare, Erlangen, Germany). To facilitate hip flexion >90°, measurements were performed in the lateral decubitus position requiring subject positioning far off-center. Maintenance of hip flexion was assured by an MR-safe belt.

BOLD-MRI is a non-invasive method using deoxygenated hemoglobin as an endogenous contrast agent which influences the relaxation time T2^*^; outcome measure is the transverse relaxation rate R2^*^ (equal to 1/T2^*^) which correlates to tissue oxygen content when confounding factors such as blood volume or hydration state are excluded. Rather than providing absolute values of oxygenation, this technique is especially useful to demonstrate relative changes in response to various interventions (13, 22). In order to standardize BOLD measurements and add a functional test, an additional maneuver had to be included; in this case, measurements were repeated after the administration of furosemide. BOLD-MRI was performed in the coronal plane using a multiple-gradient-echo sequence in a single end-expiratory breath-hold of 17 s per slice with 12 echoes equally spaced (6–52.3 ms) and the following parameters: repetition time (TR) = 65 ms, field-of-view (FOV) = 400 × 400 mm^2^, matrix = 256 × 256, slice thickness = 5 mm. All 12 images acquired from BOLD-MRI were used to estimate the R2^*^ parameters in a linear fitting model.

ASL-MRI allows quantitative perfusion measurements using magnetically labeled water as an endogenous diffusible tracer (13, 23, 24). ASL-MRI was performed using a flow-sensitive alternating inversion recovery perfusion preparation combined with a true-fast imaging with steady-state precession data acquisition according to an established protocol with pixel-based calculation of perfusion values (23, 25). Parameters were as follows: TR/echo time (TE) = 4.0/2.0 ms, slice thickness = 7 mm, matrix = 128 × 128, FOV = 360 × 360 mm^2^, inversion time = 1200 ms, averages = 30 (15 scans with inversion pulse and 15 without).

DWI allows the quantification of diffusion parameters and microperfusion yielding ADC_D_ and the perfusion fraction F_P_ (13, 22). DWI was performed with eight different b-values (0–600 s/mm^2^), two repetitions, TR of 3300 ms, TE of 56 ms, slice thickness of 5 mm, matrix of 128 × 128 and FOV of 300 × 300 mm^2^.

TOF angiography is an MR technique permitting the visualization of vascular flow without the need for contrast agents. Based on the phenomenon of flow-related enhancement of spins entering into an imaging slice, vascular anatomy can be reconstructed in a three-dimensional view.

A maximum of six regions of interest (ROIs) were analyzed in every slice (BOLD, DWI: 2–4 slices, ASL: one slice) for each of the four measurements (neutral and flexed position, before and after furosemide). ROIs were manually defined by the same blinded investigator on images handed out in a random fashion (mixed between patients and study phases). ROIs containing approximately 10 voxels were traced in the medulla and cortex. All images were co-registered facilitating comparable ROI position for all methods. Data were analyzed using in-house custom-scripts written in IDL® and MATLAB®.

### Laboratory Analyses

Serum creatinine was measured by an enzymatic creatinine assay (Roche Creatinine Plus®, Roche Diagnostics, Basel, Switzerland).

### Doppler Studies

Doppler studies were performed according to clinical routine in the outpatient clinic of the University clinic for Nephrology and Hypertension in Bern on an Acuson® 2000S device (Siemens Healthcare, Erlangen, Germany) including graft morphology, urinary outflow, and perfusion (resistance indices, flow velocities).

### Outcome Measures

The primary outcome was the change in mean medullary and cortical R2^*^ values, in medullary to cortical R2^*^ ratio (MCR R2^*^) and in R2^*^ ratio without/with (wo/w) furosemide during hip flexion compared to neutral hip position. Secondary outcomes were the changes in mean ASL perfusion values and mean F_P_ by DWI during hip flexion compared to neutral hip position, the presence of renal transplant/iliac artery kinking visualized by TOF angiography during hip flexion, the correlation of oxygenation (R2^*^, MCR R2^*^, R2^*^ ratio wo/w furosemide) and perfusion changes with the presence of functional kinking, endovascular lesions of transplant renal/iliac artery (evidenced by DUS) and with clinical parameters [age, eGFR, renin-angiotensin-aldosterone system inhibitor (RAASI) medication, calcineurin inhibitor (CNI) medication, implantation site, and donor source], as well as correlation of these clinical parameters with functional kinking.

### Statistical Analysis

Based on previous studies, we estimated that a change in R2^*^; the diffusion coefficient ADC_D_; and perfusion value of <7, 4, and 15%, respectively, could be detected in 19 patients on a significance level of 0.05 and 80% statistical power (22, 25–29), each subject serving as its own control. Quantitative variables were expressed as means with standard deviation (SD) or medians with range between minimal and maximal value. Normality testing was performed using the Kolmogorov–Smirnov test. Paired Student's *t*-test was used to compare study phases. Correlations between position-induced changes in fMRI parameters with clinical parameters were determined by Pearson and point-biserial correlation coefficient analysis as appropriate. Data were analyzed using IBM SPSS Statistics 25® and MS Office®.

## Results

### Subjects

**Figure 2:**
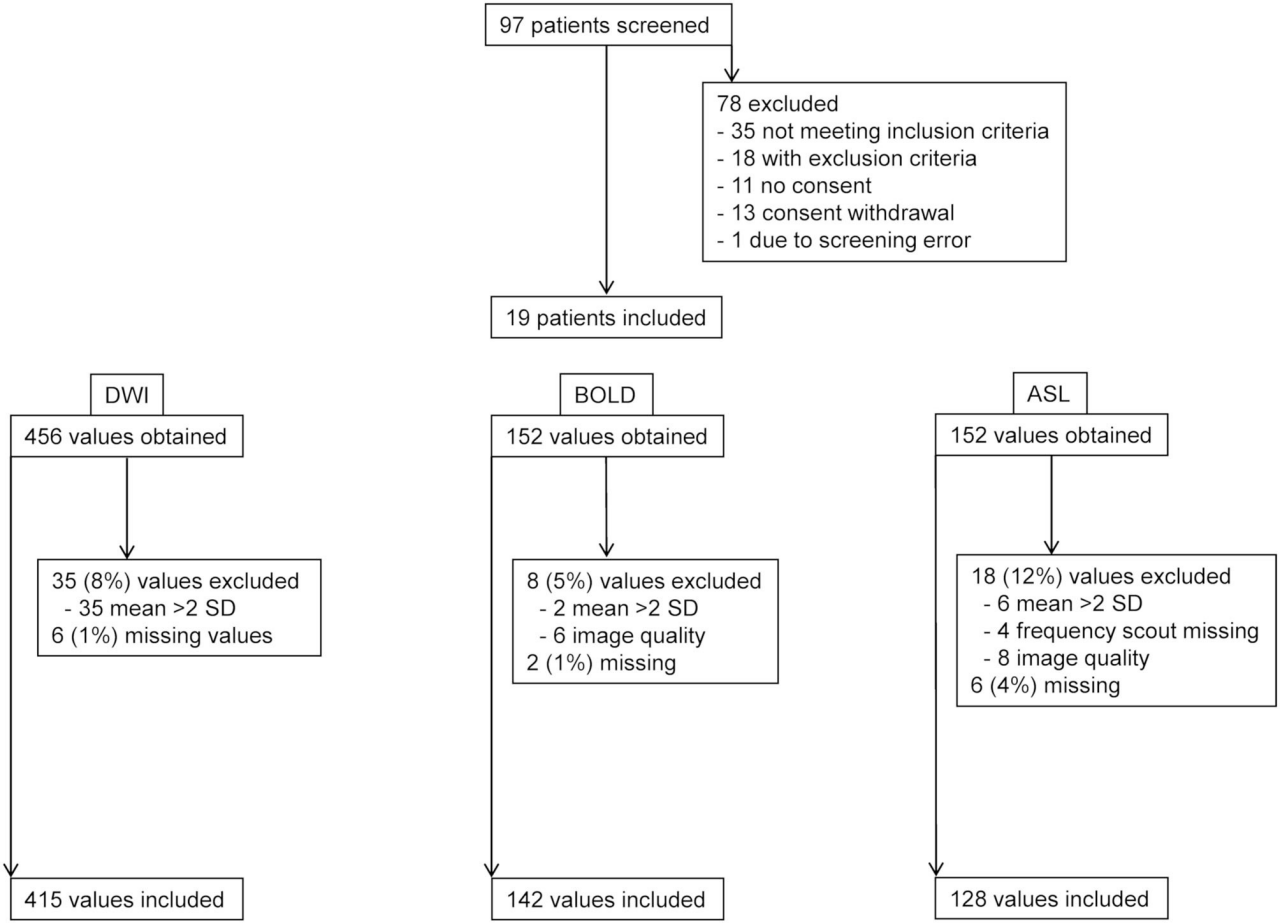
Flow chart. Patient screening and data exclusion. DWI, diffusion-weighted imaging; BOLD, blood oxygen level dependent-magnetic resonance imaging; ASL, arterial spin labeling-magnetic resonance imaging.

Nineteen kidney transplant recipients completed the study protocol (Figure 2). Baseline characteristics are shown in Table 1 and Supplementary Table 1. Subjects were mainly male Caucasian (median age 49 years) first transplant recipients (42% from living donors) with a median eGFR of 53 ml/min/1.73 m^2^. All but one subject were on antihypertensive medications including RAASI. In half the patients, vascular anastomoses involved >1 vessel and/or angioplasty. Nearly 75% of patients were on a CNI-based immunosuppressive regimen.

**Table 1:** Baseline characteristics of the subjects (*n* = 19).

Age (years)	48 ± 13 (49; 20–69)
Male gender (%)	74
Race (%)	
- Asian	16
- Caucasian	84
Living donor (%)	42
Dialysis vintage (years)	2 ± 2
Transplant episode	
- first	89
- second	11
Transplant vintage (years)	8 ± 7 (6; 0.58–20)
eGFR (ml/min/1.73 m^2^)	57 ± 19 (53; 32–102.1)
AHT medication (*n*)	2 ± 1 (0–4)
- RAASI (%)	100
CNI-based regimen (%)	74
Steroid medication (%)	63
Ipsilateral implantation (%)	11
Arteries (*n*)	1 ± 1
- with vascular intervention (%)	47
Veins (*n*)	1 ± 0
- with vascular intervention (%)	63

*Values are given as mean ± standard deviation (median; range) or as percentage of patients, as appropriate. n, number; eGFR, estimated glomerular filtration rate estimated according to chronic kidney disease epidemiology collaboration formula; AHT, antihypertensive; RAASI, renin angiotensin aldosterone system inhibitor; CNI, calcineurin inhibitor*.

### Measurement Quality

**Figure 3:**
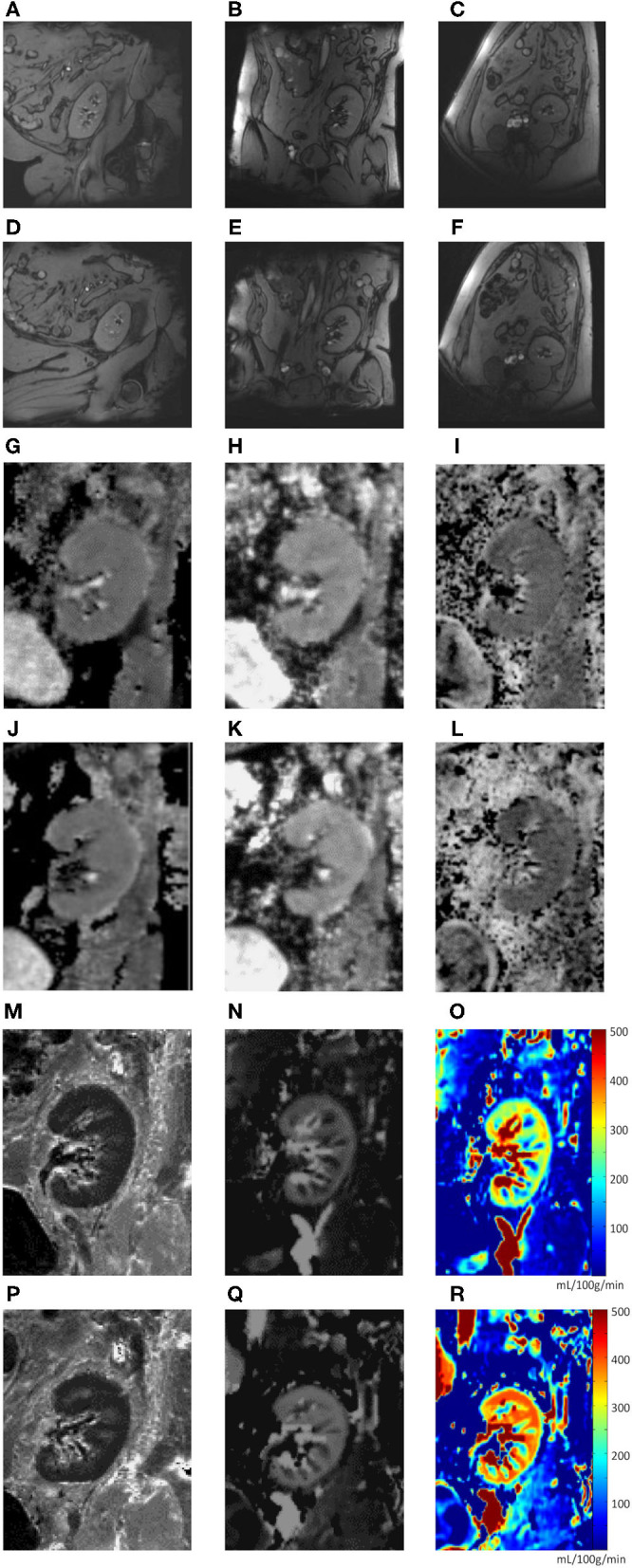
**(A–R)**. Image examples (one subject). Anatomical sequence (T1 map) during neutral hip position **(A–C)** and hip flexion **(D–F)** in sagittal **(A,D)**, coronal **(B,E)**, and transverse **(C,F)** planes. Functional MRI sequences during neutral hip position **(G–I, M–O)** and hip flexion **(J–L, P–R)**: total apparent diffusion coefficient (ADC_T_) map **(G,J)**; pure diffusion coefficient (ADC_D_) map **(H,K)**; fraction of perfusion (F_P_) map **(I,L)**; resonance transverse relaxation rate (R2*) map **(M,P)**; arterial spin labeling (ASL) map **(N,O,Q,R)**.

The MRI protocol including morphological sequences, BOLD, ASL, and DWI was successfully performed in all 19 patients in both hip positions before and in 18, 16, and 17 patients after furosemide administration, respectively. TOF angiography was performed in 16 patients during flexion after furosemide administration. Three subjects (patients 2, 4, and 12) received an incomplete dose of furosemide due to venous access problems. A mean scanning time per study phase of 15–20 min was met, resulting in an overall measurement time for all four scans <90 min including a break leaving the magnet and the time for furosemide injection. Overall visual image quality was judged good despite unusual lateral decubitus in both positions and intermediate for TOF angiography, however, without performing a formal image quality analysis (Figure 3). BOLD, ASL, and DWI-derived mean values and SD ranges were in line with previously reported values (22, 26, 30). Low SD and significant correlations between MRI parameters in hip flexion and extension as well as before and after furosemide confirmed measurement stability (Supplementary Figures 1, 2). Overall, 7–16% of values in each modality were not available for analysis due to missing data or were excluded due to poor image quality or outlier values (deviation >mean ± 2 SD) (Figure 2). Normality testing using the Kolmogorov–Smirnov test yielded *p*-values of >0.05, that is, the hypothesis of a normal distribution cannot be rejected, in 71 out of 72 tests. Solely for medullary ADC_D_ with furosemide in flexed position, the test yielded *p* = 0.04.

### BOLD-MRI

**Figure 4:**
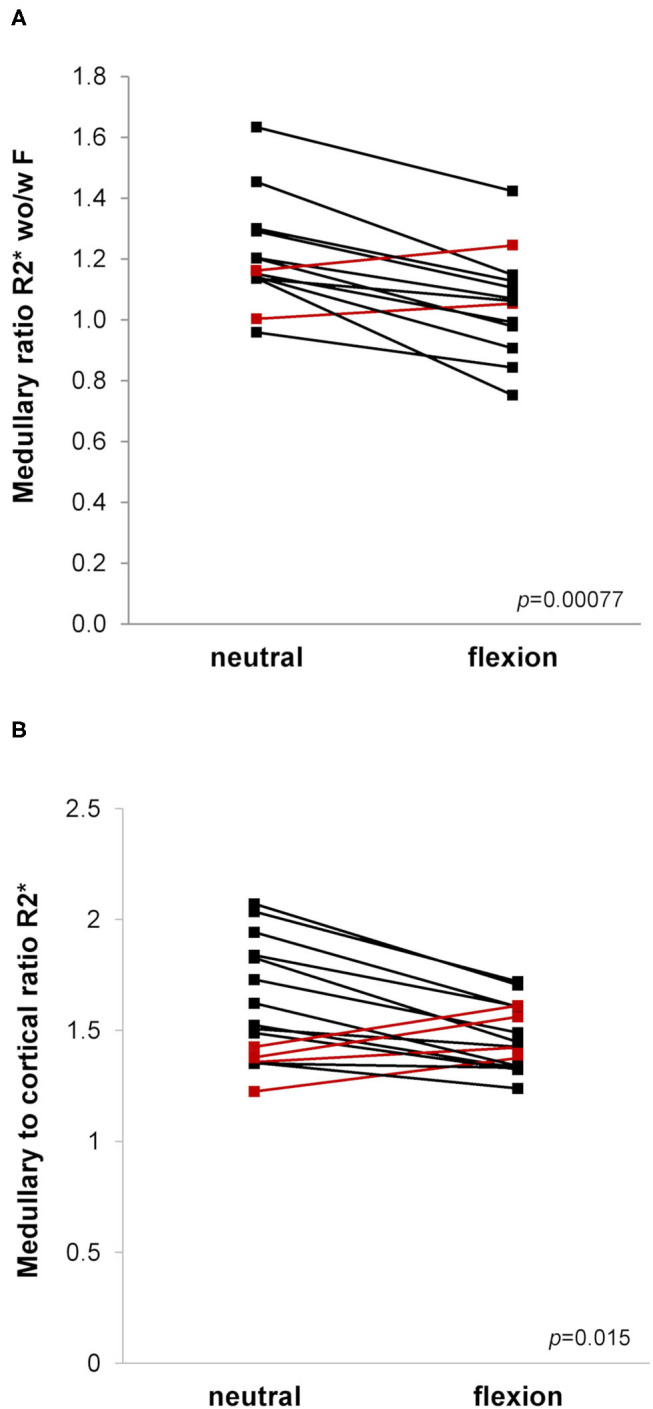
**(A,B)**. Blood oxygen level dependent (BOLD)-MRI during neutral and flexed hip position. Ladder plots showing resonance transverse relaxation rate (R2*)-derived parameters during neutral and flexed hip position: **(A)** Medullary ratio R2* without/with (wo/w) furosemide (F), *n* = 13; **(B)** Medullary to cortical ratio R2*, *n* = 16. Paired Student's *t*-test-derived p-value comparing neutral and flexed hip position (*p*). Colors denote opposite trends.

Mean medullary and cortical R2^*^ values as a marker of tissue oxygenation level (higher values meaning decreased tissue pO2) are shown in Table 2 and Figure 4. As expected, R2^*^ values were significantly higher in the medulla. Medullary R2^*^ decreased significantly after furosemide administration, as reported for native kidneys (16, 31, 32). During hip flexion, no significant changes in absolute R2^*^ values were noted. However, there was a significant decrease of the MCR R2^*^ during hip flexion suggesting a medullary oxygen redistribution, which was not observed after furosemide. The medullary R2^*^ ratio wo/w furosemide corresponding to the response to furosemide decreased highly significantly during hip flexion. In the cortex, no significant difference was observed.

**Table 2:** Functional magnetic resonance results and derived parameters.

	**wo F**		**with F**		**wo/with F**	
	**Neutral**	**Flexion**	**Neutral**	**Flexion**	**Neutral**	**Flexion**
	R2* [1/s]					
**Cortex**	17.7 ± 1.8	18.5 ± 1.4	17.3 ± 2.3	17.5 ± 2.2	1.03 ± 0.11	1.04 ± 0.10
*p*	0.14		0.77		0.53	
**Medulla**	28.3 ± 2.9	27.6 ± 3.6	24.0 ± 3.3	25.1 ± 4.1	1.22 ± 0.17	1.11 ± 0.22
*p*	0.22		0.23		0.00077	
MCR R2*	1.60 ± 0.27	1.48 ± 0.15	1.40 ± 0.20	1.44 ± 0.22	1.17 ± 0.16	1.03 ± 0.14
*p*	0.015		0.39		0.0036	
	**ASL [mL/100g/min]**					
**Cortex**	299.5 ± 60.1	332.0 ± 66.4	277.6 ± 80.6	336.7 ± 69.8	1.10 ± 0.22	0.98 ± 0.16
*p*	0.051		0.027		0.16	
**Medulla**	99.2 ± 35.5	139.9 ± 59.8	81.7 ± 22.1	147.6 ± 68.5	1.23 ± 0.59	1.14 ± 0.54
*p*	0.011		0.0052		0.41	
**MCR ASL**	0.34 ± 0.09	0.44 ± 0.15	0.32 ± 0.11	0.48 ± 0.21	1.10 ± 0.39	1.04 ± 0.42
*p*	0.026		0.033		0.37	
	ADC_D_ [*10^−5^ mm^2^/s]					
**Cortex**	208 ± 13	210 ± 13	206 ± 12	211 ± 14	1.01 ± 0.07	0.99 ± 0.05
*p*	0.5		0.163		0.42	
**Medulla**	208 ± 15	206 ± 15	202 ± 13	208 ± 15	1.03 ± 0.07	0.99 ± 0.07
*p*	0.95		0.022		0.39	
**MCR ADC**_**D**_	1.01 ± 0.06	0.99 ± 0.05	0.98 ± 0.05	0.99 ± 0.07	1.03 ± 0.07	1.01 ± 0.09
*p*	0.62		0.77		0.8	
	**F_P_ [%]**					
**Cortex**	11.7 ± 3.3	12.9 ± 3.6	11.3 ± 4.2	11.1 ± 4.9	1.06 ± 0.42	1.34 ± 0.50
*p*	0.5		0.34		0.076	
**Medulla**	5.9 ± 4.6	7.4 ± 4.5	10.1 ± 3.6	10.7 ± 4.0	0.64 ± 0.48	0.80 ± 0.64
*p*	0.51		0.78		0.66	
**MCR F**_**P**_	0.60 ± 0.51	0.54 ± 0.27	0.93 ± 0.35	1.11 ± 0.62	0.72 ± 0.52	0.67 ± 0.53
*p*	0.63		0.16		0.6	

Values are given as mean ± standard deviation. p-values are calculated according to Student's t-test. Wo, without; F, furosemide; neutral/flexion denotes hip position; R2^*^, transverse relaxation rate; MCR, medullary to cortical ratio; ASL, arterial spin labeling; ADC_D_, pure diffusion coefficient; F_P_, fraction of perfusion.

### ASL-MRI

**Figure 5:**
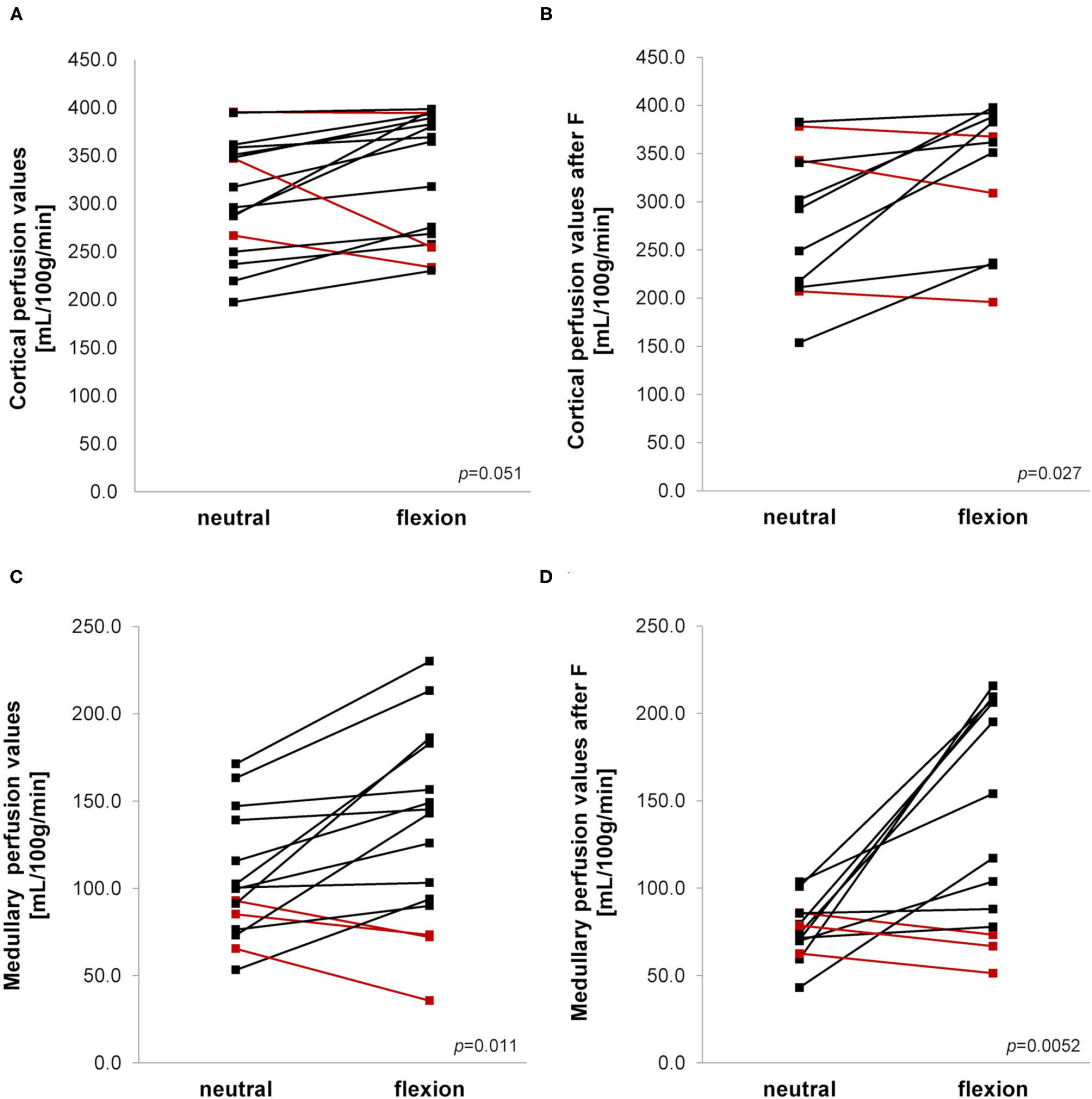
**(A–D)** Arterial spin labeling-MRI during neutral and flexed hip position. Ladder plots showing perfusion values measured in cortex **(A,B)** and medulla **(C,D)** without **(A,C)** and after **(B,D)** furosemide (F) administration during neutral and flexed hip position. **(A)** *n* = 16; **(B)** *n* = 11; **(C)** *n* = 15; **(D)** *n* = 13. Paired Student's *t*-test-derived *p*-value comparing neutral and flexed hip position (*p*). Colors denote opposite trends.

Mean medullary and cortical perfusion values obtained by ASL-MRI indicating macro-perfusion are shown in Table 2 and Figure 5. There was a consistent increase in mean perfusion values during hip flexion reaching statistical significance in medullary measurements as well as in the cortex after furosemide injection. The perfusion increase was stronger in the medulla than in the cortex resulting in a significantly higher medullary to cortical-ratio during hip flexion.

### DWI

Mean medullary and cortical values for the diffusion coefficient ADC_D_ as a marker of pure diffusion are shown in Table 2. As expected, no change induced by hip flexion was observed in the cortex. In medulla, ADC_D_ values were significantly higher during hip flexion after administration of furosemide. Perfusion fraction F_P_, a parameter of microperfusion, showed no significant changes dependent on hip position (Table 2).

### TOF Angiography

TOF angiography did not identify kinking phenomena during hip flexion (data not shown).

### Correlation to Clinical Parameters

To investigate determinants of position-induced oxygenation and perfusion changes, correlation testing was performed between fMRI and selected clinical parameters (age, transplant vintage, eGFR, donor source, and CNI use). To reduce the number of comparisons, only significant changes between positions were tested. Two significant correlations were found:

First, in BOLD, the change in MCR R2^*^ from neutral to flexed position without furosemide correlated positively with eGFR (R = 0.52, *p* = 0.039). This might indicate a stronger cortical R2^*^ increase (oxygenation decrease) during flexion in patients with reduced graft function.

Second, in ASL, the increase in cortical perfusion from neutral to flexed position without furosemide correlated negatively and significantly with age (R = −0.52, *p* = 0.041) and transplant vintage (R = −0.745, *p* = 0.001) as well as with CNI use after furosemide (r_pb_ = −0.604, *p* = 0.049).

### Duplex Studies

DUS performed in all subjects within 6 months of the study day ruled out renal and iliac arterial or venous flow restrictions; in one patient, the arterial anastomosis could not be visualized.

## Discussion

The main findings of this study are the following: (1) hip flexion induces a consistent increase in renal perfusion as measured by ASL; (2) hip flexion causes a redistribution of renal tissue oxygenation with a significant decrease of the medulla/cortex ratio R2^*^. This effect on renal oxygenation was not observed after furosemide. (3) Hip flexion diminishes the increase in oxygenation after furosemide. At last, our study demonstrates for the first time the feasibility and reliability of a multiparametric functional MRI protocol with examination of kidney graft oxygenation and perfusion during two different body positions in a single session.

Our initial hypothesis was that hip flexion would lead to an instant and temporary reduction of graft perfusion associated with a decrease in graft oxygenation. To our surprise, perfusion values measured by ASL-MRI consistently increased during hip flexion. ASL technique involves the magnetic labeling of inflowing blood with the creation of a subtraction image of the kidney with and without labeling (25). In models of renal ischemia due to renal artery stenosis, decreased perfusion values have been described with this technique (33). There are several possible explanations for the unexpected increase in graft perfusion upon hip flexion.

The first is an increase in arterial inflow due to the partial obstruction distally to the graft leading to increased flow through the external and internal iliac artery. In line with this hypothesis, maximal hip flexion has been shown to induce a shortening, bending, and twisting of iliac arteries in healthy subjects (12, 34). In patients with leg amputation, significant changes in arterial hemodynamics proximal to the amputation have been observed, characterized by early return of reflected waves and an increase in shear stress (34). In patients with traumatic lower limb amputation, these changes have been associated with an increased risk for cardiovascular diseases including aortic aneurysms.

A second hypothesis is that hip flexion causes an increase in arterial blood pressure secondary to a global increase in systemic vascular resistance. Analogously, the squatting position classically adopted by patients with cyanotic heart disease leads to blood pressure elevations by increasing venous return and systemic arterial resistance. Yet, this effect might be restricted to the standing position (35, 36). However, in our experiments, the effect was even greater after furosemide administration, which should lead to a decrease rather than increase in systemic blood pressure.

The increase in renal perfusion could also represent a counter-regulatory mechanism secondary to the changes in tissue oxygenation. Indeed, the scan order putting BOLD before ASL sequences might explain inverse trends in BOLD and ASL values. Thus, in a recent study, sympathetic stimulation by handgrip exercise has been reported to decrease renal artery flow and increase medullary oxygenation measured by BOLD-MRI. A decreased reabsorptive workload due to reduced distal sodium delivery was hypothesized by the authors (37).

Venous outflow obstruction with backflow to the graft during hip flexion is still another mechanism whereby hip flexion could increase graft perfusion. Venous obstruction might actually explain both the increased renal perfusion and the reduced oxygenation response to furosemide. In this situation, the marked prolonged position change might have overcome the hurdle of venous valves. The hydration protocol inducing a larger plasma volume may have increased this effect. Similarly, the two subjects with ipsilateral graft implantation (with a risk of arterio-venous crossing) showed a particularly reduced perfusion ratio neutral/flexed hip position possibly pointing to outflow obstruction. Importantly, potential changes in arterial inflow may have been masked in this case. Thus, in this view, the positive correlation between age, transplant vintage and CNI use on one hand and the cortical perfusion ratio neutral/flexed hip position on the other may be explained by a reduced arterial component of overall flow. However, this hypothesis remains speculative.

The perfusion increase in the cortex and medulla during hip flexion after furosemide is augmented by the observed (albeit non-significantly) lower baseline perfusion values during neutral hip position after furosemide compared to the values before the administration of furosemide. This is in line with previous microelectrode measurements in rats and ASL-MRI measurements in humans (38, 39).

In accordance with our initial hypothesis, hip flexion resulted in changes of renal tissue oxygenation characterized by a significant decrease in the medullary-to-cortical R2^*^ ratio, suggesting a redistribution of intrarenal tissue oxygenation toward the medulla. The decrease of MCR R2^*^ during hip flexion correlated with lower eGFR values. MCR R2^*^ has previously been defined as the normalized intrarenal oxygen bioavailability (40). The significant decrease in MCR R2^*^ during hip flexion points to a decreased cortical oxygen availability or medullary redistribution similar to that described in CKD and vascular allograft rejection (40–43). However, after furosemide administration this effect was not reproduced. This may be due to the strong medullary R2^*^ decrease induced by furosemide which might have outweighed the difference induced by the positional change. When considering absolute R2^*^ values showing no significant differences between both body positions in this study, the confounding factor of the regional blood volume has also to be discussed. Indeed, vasoconstriction might lead to only minor R2^*^ increases and even R2^*^ decreases despite reduced oxygen delivery due to a decreased blood volume fraction (44).

Another finding of our study supporting the initial hypothesis is that the improvement in medullary oxygenation observed upon administration of furosemide under normal conditions was attenuated during hip flexion. As shown previously in native kidneys, furosemide decreases R2^*^, reflecting an increased tissue oxygenation. This effect is thought to result from decreased oxygen consumption through Na-K-2Cl-cotransporter inhibition (16, 31, 32, 38, 45). The blunted effect of furosemide during hip flexion could be due either to a resistance to furosemide caused by an acute tubular dysfunction or to increased sodium reabsorption secondary to an activation of the renin-angiotensin-aldosterone system, although most of the patients were on a blocker of the renin-angiotensin system. It may also be the consequence of a relative reduction of oxygen supply during hip flexion. Our findings are in accordance with reports on reduced BOLD response to furosemide without effect on absolute R2^*^ values in acute and chronic kidney disease states, in chronic arterial hypertension and during aging (17, 42, 45–47). Furosemide administration has indeed been used as a test of renal functional reserve enhancing the sensitivity of BOLD-MRI similarly to furosemide stress test in acute kidney injury (48).

Our study has several limitations: high-field-strength MRI does not allow imaging in an upright position; therefore, the sitting position had to be simulated in lateral decubitus excluding the additional influence of gravity potentially operating during the usual sitting position. Blood pressure was not measured simultaneously to evaluate the influence on perfusion values. For ethical reasons, contrast-enhanced angiography was not performed to evaluate for the presence of arterial kinking of iliac arteries and preexisting vascular lesions. Instead, TOF angiography and DUS were used enabling the exclusion of renal artery stenosis or arterial abnormalities in these patients. Another potential limitation is the absence of a measure of graft function during hip flexion. The MRI protocol was too complex to perform simultaneous functional measurements, but this aspect would deserve additional studies. For security reasons, patients with foreign material such as vascular stents and no previous 3T-MRI were excluded. Thus, we may have selected a less atherosclerotic population. The number of subjects in this proof of principle-study was limited. Due to the number of cases, only univariate analysis of clinical associations was performed. Lastly, correction for multiple comparisons was not carried out in this exploratory analysis of clinical correlations.

In conclusion, this physiological proof of principle-study suggests for the first time that redistribution of oxygenation and functional hypo-oxygenation of renal transplants may occur depending on hip position. Whether this phenomenon contributes to the development of chronic fibrosis and ultimately to graft dysfunction is not known. However, one could hypothesize that besides immunological and known non-immunological factors, recurrent graft hypo-oxygenation might also play a role in the long-term loss of kidney grafts. The potential implication of our observations could be a recommendation to avoid a prolonged sitting position in kidney graft recipients and to favor exercises without major sustained hip flexion, such as rowing. Further research is required to assess the functional and/or histological impact of our observation in patients showing a pathologic response during this maneuver.

## Data Availability Statement

The raw data supporting the conclusions of this article will be made available by the authors, without undue reservation.

## Ethics Statement

The studies involving human participants were reviewed and approved by Ethics commitee of the Canton of Bern, Switzerland (protocol number 2181, approval number 042/12). The patients/participants provided their written informed consent to participate in this study.

## Author Contributions

LYM participated in research design, performance of the research, data analysis, and writing of the manuscript. MS participated in research design, performance of the research, data collection, and revision of the manuscript. FN participated in data analysis and the revision of the manuscript. DT participated in data analysis and the revision of the manuscript. GD participated in data analysis. PM contributed the arterial spin labeling–magnetic resonance imaging sequence and participated in the revision of the manuscript. MB participated in the writing and revision of the manuscript. BV participated in the research design, performance of the research, and revision of the manuscript. PV participated in the research design, performance of the research, data collection, data analysis, and revision of the manuscript. All authors contributed to the article and approved the submitted version.

## Conflict of Interest

The authors declare that the research was conducted in the absence of any commercial or financial relationships that could be construed as a potential conflict of interest.

## Publisher's Note

All claims expressed in this article are solely those of the authors and do not necessarily represent those of their affiliated organizations, or those of the publisher, the editors and the reviewers. Any product that may be evaluated in this article, or claim that may be made by its manufacturer, is not guaranteed or endorsed by the publisher.

## Abbreviations

ADC_D_: diffusion coefficient
ASL: arterial spin labeling
BOLD: blood oxygenation level dependent
CNI: calcineurin inhibitors
CKD-EPI: chronic kidney disease epidemiology collaboration
DUS: duplex ultrasound scan
DWI: diffusion-weighted imaging
eGFR: estimated glomerular filtration rate
fMRI: functional magnetic resonance imaging
FOV: field of view
F_P_: perfusion fraction
IFTA: interstitial fibrosis and tubular atrophy
MCR R2^*^: medullary to cortical R2^*^ ratio
MR: magnetic resonance
MRI: magnetic resonance imaging
RAASI: renin-angiotensin-aldosterone system inhibitors
R2^*^: transverse relaxation rate
ROI: region of interest
SD: standard deviation
TE: echo time
TOF: time of flight
TR: repetition time
wo/w: without/with.
