# Supplementary material for: Hip Position Acutely Affects Oxygenation and Perfusion of Kidney Grafts as Measured by Functional Magnetic Resonance Imaging Methods—The Bent Knee Study
Source: Front Med (Lausanne). 2021 Aug 10;8:697055. doi: 10.3389/fmed.2021.697055 (PMC8384256; doi:10.3389/fmed.2021.697055)
Supplement: Supplementary file 1 [file Image_1.pdf]

Supplementary Figure 1

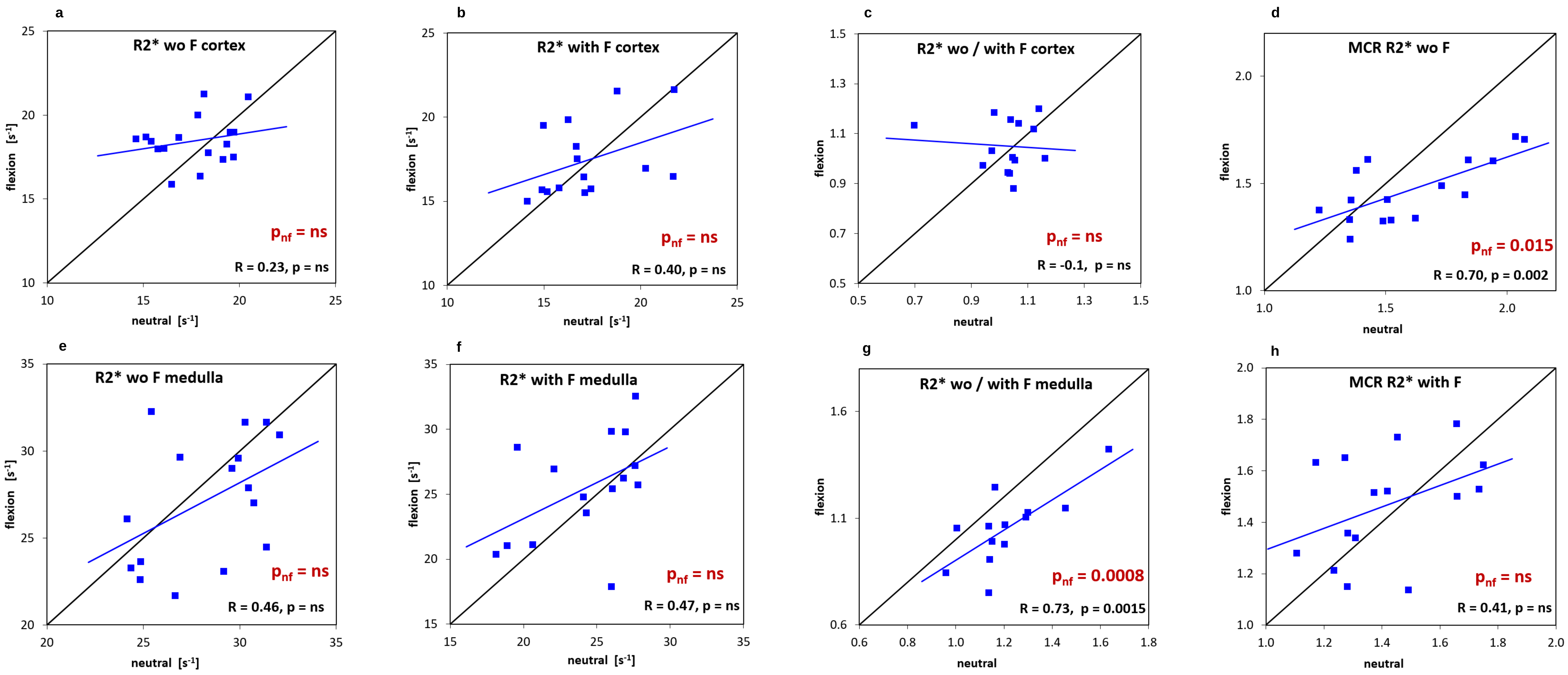

Supplementary Figure 1. Blood-oxygen-level-dependent (BOLD)-MRI during neutral and flexed hip position. Scatter plots showing correlation between neutral and flexed hip position. Resonance transverse relaxation rate

(R2*) values measured in cortex (a-c) and medulla (e-g) without (wo) (a, e) and after (b, f) furosemide (F) administration. Ratio R2* wo/with furosemide in cortex and medulla (c, g). Medullary to cortical ratio (MCR) (d, h). p_{nf} (p-value for comparison of neutral and flexed hip position, students t-test); R (Pearson correlation coefficient); p (p-value for correlation of neutral and flexed hip position), ns (not significant).
