# Supplementary material for: Hip Position Acutely Affects Oxygenation and Perfusion of Kidney Grafts as Measured by Functional Magnetic Resonance Imaging Methods—The Bent Knee Study
Source: Front Med (Lausanne). 2021 Aug 10;8:697055. doi: 10.3389/fmed.2021.697055 (PMC8384256; doi:10.3389/fmed.2021.697055)
Supplement: Supplementary file 2 [file Image_2.pdf]

# Supplementary Figure 2

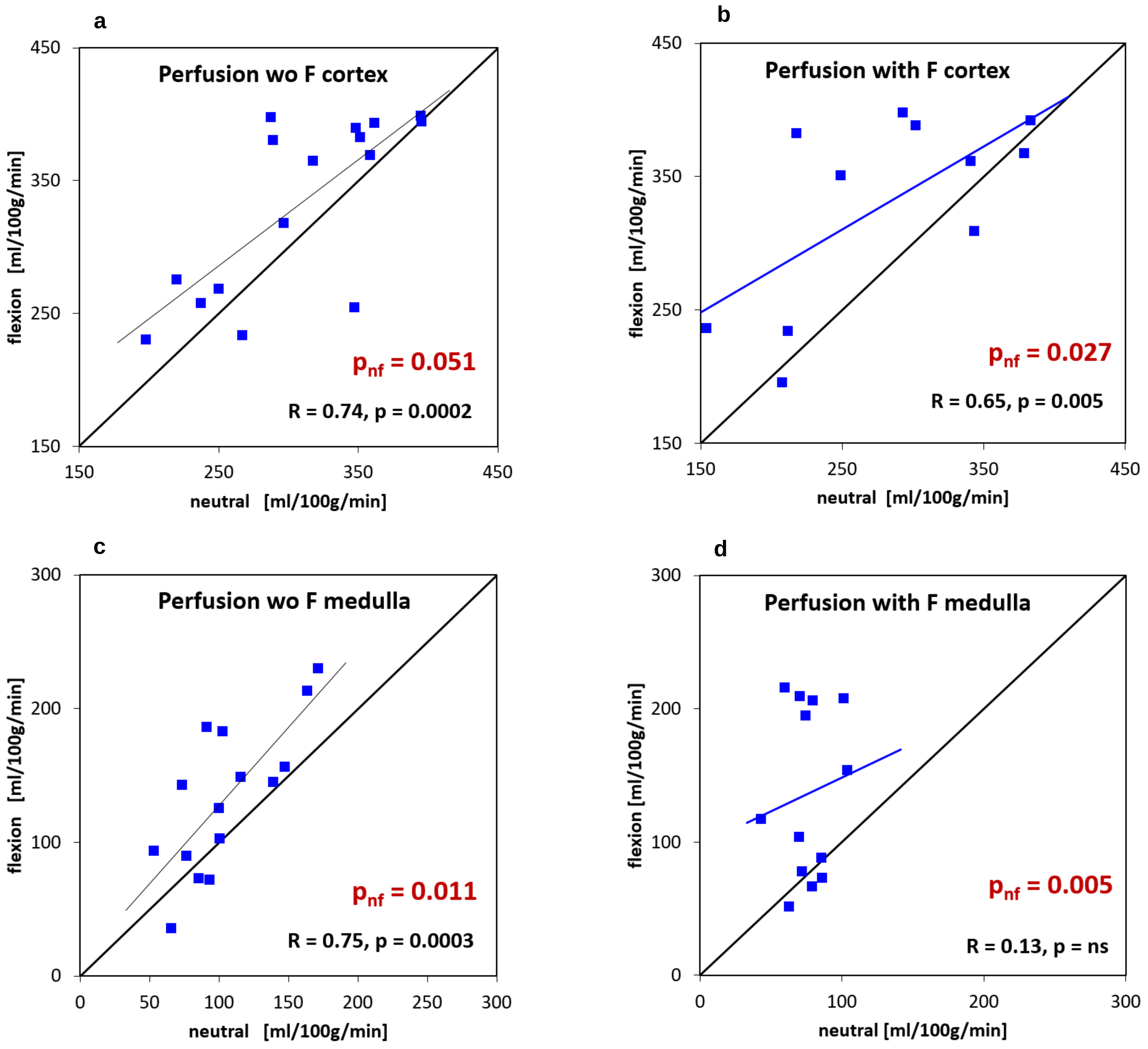


**Supplementary Figure 2 a-d. Arterial spin labeling-MRI during neutral and flexed hip position.** Perfusion values measured in cortex (a, b) and medulla (c, d) without (wo) (a, c) and after (b, d) furosemide (F) administration. $p_{nf}$ (p-value for comparison of neutral and flexed hip position, students t-test); R (Pearson correlation coefficient); p (p-value for correlation of neutral and flexed hip position), ns (not significant).
