# Supplementary material for: Hip Position Acutely Affects Oxygenation and Perfusion of Kidney Grafts as Measured by Functional Magnetic Resonance Imaging Methods—The Bent Knee Study
Source: Front Med (Lausanne). 2021 Aug 10;8:697055. doi: 10.3389/fmed.2021.697055 (PMC8384256; doi:10.3389/fmed.2021.697055)
Supplement: Supplementary file 3 [file Table_1.pdf]

Supplementary Table 1

Supplementary Table 1. Baseline characteristics of the subjects

	Age (yrs)	Gender	Race	Donor source	Dialysis vintage (yrs)	Tx episode	Tx vintage (yrs)	eGFR-CKD-EPI (ml/min/1.73 m^2)	AHT medication (n)	RAASI	CNI	Steroids	Site of origin	Implant. site (FI)	Arteries (n)	Veins (n)
P1	65	m	c	living	0.92	1	6	80	3	1	1	1	R	L	2	1
P2	61	m	c	deceased	3.5	1	3	42	4	1	0	1	R	L	2+patch	2 + patch
P3	47	m	c	living	0.17	1	7	67	4	1	1	1	R	L	1	1
P4	42	m	c	deceased	1	1	20	35	1	1	1	0	L	R	2+patch+ lig	1+patch+ lig
P5	32	f	a	deceased	4.6	2	6	56	1	1	1	1	R	L	1	1+ *
P6	56	m	a	deceased	1.75	1	8	40	3	1	1	0	L	R	1+patch	1+patch
P7	33	m	c	living	0	1	0.58	70	0	0	1	1	R	L	1	1
P8	41	m	a	deceased	2	1	11	53	2	1	1	0	L	R	1+patch	1+patch
P9	64	m	c	deceased	2.17	1	11	64	2	1	1	1	L	R	1+patch	1+patch
P10	50	m	c	deceased	6.3	1	5	89	2	1	0	1	R	L	1+patch	1+patch
P11	49	m	c	living	1.8	1	0.67	57	1	1	1	1	L	R	1	1
P12	69	f	c	deceased	1.5	1	26	32	4	1	1	1	R	L	2	1
P13	50	m	c	living	0.5	1	8	87	2	1	1	0	L	R	1	1
P14	39	m	c	living	0	1	9	39	1	1	0	1	R	L	1	1
P15	62	f	c	deceased	3.92	2	4	46	3	1	0	1	R	R	1+patch	1+patch
P16	20	f	c	deceased	0	1	0.58	102.1	1	1	1	1	L	R	1+patch	1+patch
P17	50	f	c	deceased	1.3	1	13	43	4	1	0	0	R	L	2+patch	1+patch
P18	36	m	c	living	0.3	1	2	39	2	1	1	0	L	R	2	1+patch
P19	47	m	c	living	1	1	3	45	2	1	1	0	R	R	1	1+patch
Mean/SD	48±13	-	-	-	2±2	-	8±7	57±20	2±1	-	-	-	-	-	-	-

P (patient); Yrs (years); m (male); f (female); c (Caucasian); a (Asian); Tx (transplantation); eGFR (estimated glomerular filtration rate); CKD-EPI (chronic kidney disease epidemiology formula); AHT (antihypertensive); n (number); RAASI (renin angiotensin aldosteron system inhibitor); CNI (calcineurin inhibitor); R (right); L (left); Implant. (Implantation); FI (fossa iliaca); * + vena cava elongation plasty, lig (ligature);SD (standard deviation).
